# Exploring Agreement in Voice Acoustic Parameters: A Repeated Measures Case Study Across Varied Recording Instruments, Speech Samples, and Daily Timeframes

**DOI:** 10.3390/acoustics7010006

**Published:** 2025-01-22

**Authors:** Lady Catherine Cantor-Cutiva, Adrián Castillo-Allendes, Eric James Hunter

**Affiliations:** 1 Department of Communicative Sciences and Disorders, University of Iowa, Iowa City, IA 52242, USA; 2 Department of Audiology and Speech-Language Pathology, East Tennessee State University, Johnson City, TN 37614, USA; 3 Department of Communicative Sciences and Disorders, Michigan State University, East Lansing, MI 48824, USA

**Keywords:** voice, voice acoustic parameters, microphone, accelerometer, repeated measures

## Abstract

**Aims:**

The aim was to assess the agreement between microphone-derived and neck accelerometer-derived voice acoustic parameters and their associations with recording moments and speech types.

**Methods:**

Using simultaneous recordings, a 7-week study on a single individual was conducted to reduce intersubject variability. Agreement was assessed using Bland–Altman plots, and associations were examined with generalized estimating equations.

**Results:**

Bland–Altman plots showed no significant bias between microphone (MIC) and accelerometer (ACC) measurements for alpha ratio, CPP, PPE, SPL SD, fundamental frequency (fo) mean, and SD. Speech type and measurement timing were significantly associated with alpha ratio, while the instrument was not. Microphone measurements resulted in slightly lower CPP compared to the accelerometer, while reading samples yielded higher CPP compared to vowel productions. PPE, SPL SD, and fo mean showed significant associations with speech type, based on univariate analysis. Microphone measurements yielded a statistically smaller fo SD compared to the accelerometer, while reading productions had a larger fo SD than vowel productions.

**Conclusion:**

Fundamental frequency, alpha ratio, PPE, and SPL SD values were robust, regardless of the instrument used, suggesting the potential use of accelerometers in less-controlled environments. These findings are crucial for enhancing confidence in voice metrics and exploring efficient clinical assessment protocols.

## 1. Introduction

During voice therapy assessments, vocal health practitioners use a wide range of in-clinic instrumentation, protocols, and metrics to determine the efficiency of intervention strategies. These tools include speech materials, like sustained vowel productions and readings [1,2], which may not represent real-life voice use. This can hinder a comprehensive understanding of voice performance. To address this, “ecological voice assessments” emerged as a valuable complement [3,4]. These assessments involve recording actual voice use in daily contexts, like an occupational setting, offering a more holistic and realistic picture of vocal demands. Moreover, with the latter in particular, clinicians gain the ability to not only track actual vocal use but also to sample the impact of therapy on an individual’s daily communicative environment. This capability extends to remote therapy and assessment, scenarios beyond the scope of isolated simulated vocal tasks employed in traditional clinical settings. Nevertheless, moving towards more ecologically valid voice assessments introduces additional uncertainty and variability that already exist in assessing voice production.

Speech and voice acoustic parameters can exhibit significant variability due to numerous influencing factors. The natural inter- and intrasubject variabilities in voice parameters are commonly mitigated by controlling the vocal demand (e.g., communication partner, environment noise, vocal goal, voice content) and/or by increasing the number of samples and repetitions [5,6]. For example, the voice or speech content, such as reading passages versus vowel production, has an impact on parameters, even for the same individual [7,8]. The context or time of day of voice production, such as capturing voice use before and after specific interventions or daily activities, also illustrates the dynamic nature of vocal production and vocal health [5]. Understanding the sources of variability, which acoustic parameters are less sensitive to variability, and how to mitigate such variability, when possible, are important for a holistic understanding of voice use and ecological voice assessment [9,10].

In pursuit of capturing authentic voice use outside clinical settings while trying to mitigate variability, technological advancements have led to integrating various types of transducers in ambulatory voice monitoring devices. These instruments are key in surpassing the limitations of traditional in-clinic assessments, offering a window into more natural vocalization patterns within the clinic and in more ecologically relevant environments. Nonetheless, while ambulatory voice monitoring, or voice dosimetry, has been used in various research studies in real-world situations, it has not yet become clinically viable. Using more ecologically valid conditions for vocal assessment requires a different approach to vocal recording and quantification [11,12]. Where most clinical assessments call for highly prescribed vocal production instructions or controlled communication settings, ecological monitoring of the voice inherently has much less control and should be sensitive to patient privacy, capable of recording longer samples, clinically sustainable and efficient, and robust to a variety of situations (e.g., communication partners and environmental noises). To address these requirements, previous attempts [13,14] have used some combination of open-air microphones (MIC, both omnidirectional and directional), neck-attached contact microphones (MIC), and neck-attached accelerometers (ACC).

In ecological voice monitoring, the primary value of contact microphones and ACCs is that their response to physical vibrations at the sampling site makes them less sensitive to environmental noise artifacts [15,16]. However, this feature comes with the liability of reduced spectral sensitivity [17,18], which may affect derived voice and speech acoustic parameters. In contrast, open-air microphones have a better spectral response for recording voice and speech, but the voice and speech of interest will be contaminated with a variety of acoustic artifacts (e.g., voice of other talkers, reverberation, static, and transient background noise), which can also significantly impact derived acoustic parameters.

Accelerometers, microphones, and contact microphones have been compared for their ability to measure vocal fundamental frequency and other vocal parameters [13,19]. As mentioned above, each type of sensor has its advantages and drawbacks; nevertheless, transducers like the neck-worn ACC have been shown to be effective complements to acoustic recordings in the assessment and monitoring of vocal function. This effectiveness, however, is nuanced by the inherent limitations of each sensor type, especially when considering the environmental impacts affecting acoustic measurements [20,21]. For example, the transducer characteristics of the ACC, affecting spectral sensitivity, have traditionally limited such devices to measuring a narrow set of voice parameters, focused on voice fundamental frequency, and estimated vocal sound pressure levels and their derivatives [22–24]. These reduced parameters have limited the adoption of devices like the ACC in the natural environment or in a noisy clinic, which impacts their usefulness in supporting clinical decision-making. Considering factors such as intrasubject variability, differences in transducers, environmental impacts, and types of vocal content all affect the reliability and validity of voice acoustic measurements [25,26]. This information is needed to better understand the evolution of voice disorders, as well as the effectiveness of intervention strategies.

To address these issues, we conducted a 7-week follow-up study of one occupational voice user with current voice complaints with two aims: [1] to assess the agreement between microphone-derived and neck accelerometer-derived voice acoustic parameters and [2] to evaluate whether the microphone-derived and neck accelerometer-derived voice acoustic parameters show similar associations with the moment of the recording (pre vs. post) and type of speech production (reading vs. vowel). This study aims to test two specific hypotheses. First, we hypothesize that there will be a significant correlation between acoustic parameters derived from microphones and those derived from neck accelerometers. Second, we hypothesize that both microphone-derived and neck accelerometer-derived parameters will exhibit similar associations with the timing of the recording (pre- vs. post-intervention) and the type of speech production (reading vs. vowel). These hypotheses are designed to evaluate the reliability and applicability of neck-worn accelerometers in capturing voice data in real-world settings, thereby informing their potential use in clinical voice assessments. The results will enable a more informed selection of acoustic parameters in voice care and promote the intentional use of accelerometers in voice assessment.

## 2. Materials and Methods

These procedures were designed to simulate a clinically useful vocal monitoring situation with greater ecological validity than common protocols. The device used was portable for clients, instructions were simple for most voice clients to follow, and recordings were conducted by clients in a natural, repeatable setting.

### 2.1. Participant and Study Design

A male college professor (48 years) who possessed experience and knowledge in voice production was recruited. The participant disclosed current moderate hoarseness with a history of voice disorders, laryngopharyngeal reflux, and a high occupational vocal load. Additionally, the participant indicated generally good vocal hygiene practices, with no reported use of tobacco or alcohol. This study was conducted with the approval of the Michigan State University Human Research Protection Program (IRB 13–1149).

For this longitudinal/test–retest study, following the diagnosis of the bilateral sulcus, the male participant was enrolled in a 7-week voice observation program, where there was regular use of water resistance therapy (WRT), a form of semi-occluded phonation. Further details of the WRT and its results may be found in a previously published paper [5].

### 2.2. Instrument and Procedures

**Figure 1.**
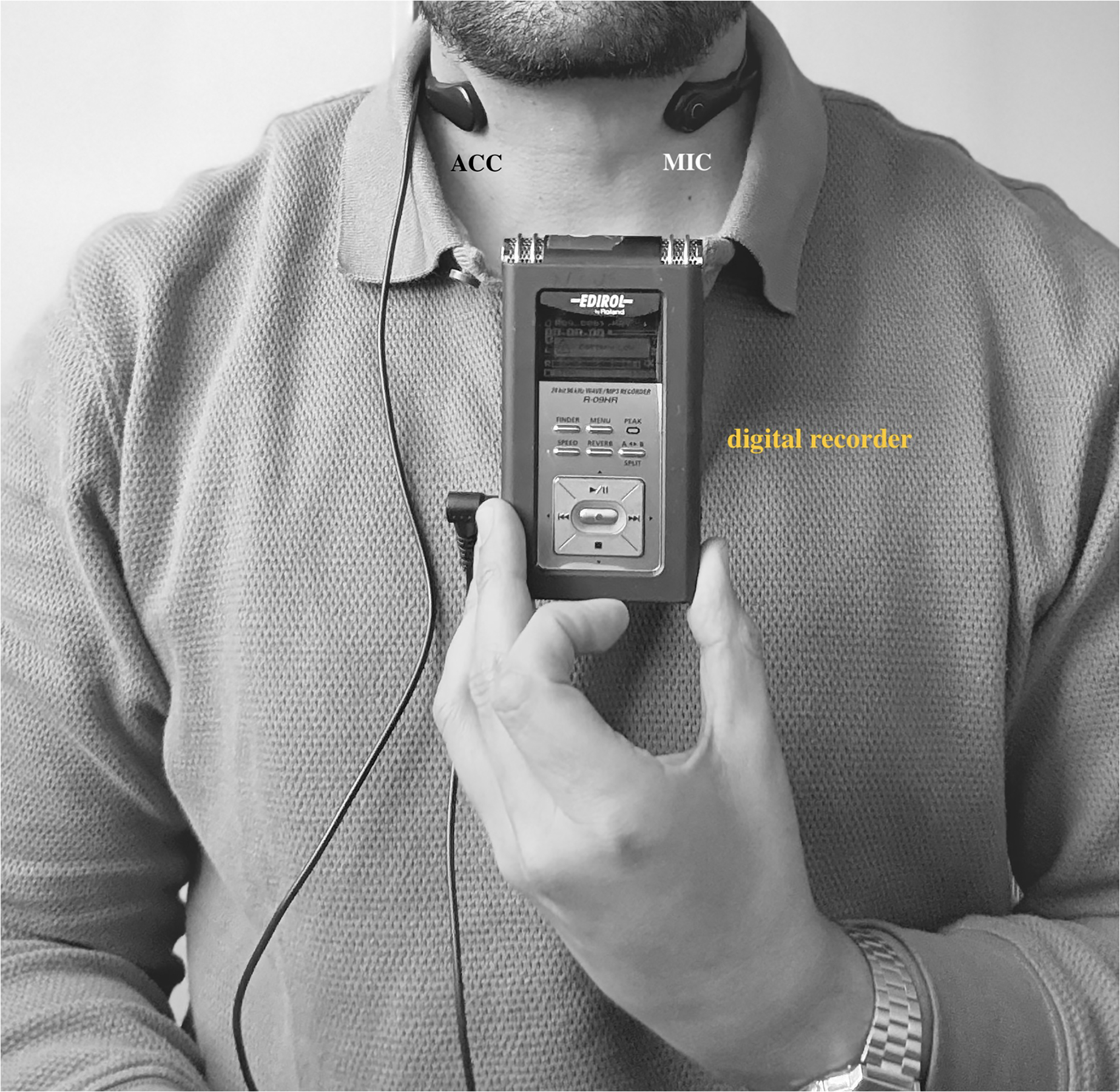
Setup utilized for recording, showing the VoxLog neck collar (SonVox) with an accelerometer (ACC) and microphone (MIC) positioned on the anterior region of the neck, connected to a Roland R-05 digital recorder.

Recordings were performed in a private office (220 sf) with low background noise levels (Leq 27.2 dbA) and short reverberation time (T20 = 0.21 s), with minimal external noise and consistent acoustics. This controlled setting helped to maintain a stable recording environment. In addition, both the microphone and accelerometer were placed according to standardized procedures to ensure consistent data collection. This included maintaining a fixed distance from the sound source and using calibrated equipment. By implementing these measures, we ensured that environmental variables were effectively controlled or accounted for, thereby enhancing the validity of our findings. As shown in Figure 1, all recordings were conducted using the neck collar from the VoxLog (SonVox) voice monitoring system, along with a digital recorder (Roland R-05) at common voice/speech recording parameters (41,000 Hz, 16-bit, wav). The VoxLog neck collar, which is worn about the neck (Figure 1), consisted of an accelerometer and a factory-calibrated air microphone [14,27]. All voice recordings included a steady sustained vowel [a:] produced three times at a comfortable pitch and loudness and the first paragraph of the “Rainbow Passage” (reading) [28]. These were performed twice each session, before and after the WRT, at various times of the day [5].

We selected the Rainbow Passage and the sustained vowel [a:] as our speech samples, due to their widespread use and acceptance in voice research and clinical assessments. The Rainbow Passage is a phonetically balanced text that provides a comprehensive representation of various speech sounds, making it an ideal choice for assessing voice and speech parameters. However, to ensure consistency and manageability in our analysis, we focused on the first two sentences of that text. This decision was based on the need to balance the richness of the speech sample with the practical constraints of data processing and analysis. The sustained vowel [a:] provides a stable and consistent phonatory task that allows for the reliable measurement of fundamental frequency and other acoustic parameters. The simplicity and consistency of the sustained phonation make it a valuable tool for comparing voice parameters across different conditions and devices. Further, by nature of the reading sample, there would be more high-frequency energy from consonants (e.g., fricatives, sibilance, plosives), voice onset and offset instability and noise, and more change in the speech fundamental frequency due to common linguistic variabilities. By selecting these speech samples, we aimed to leverage their established validity and reliability in voice research while ensuring that our analysis remained focused and manageable.

### 2.3. Acoustic Analysis

In total, 340 audio recordings were made during thirty-two non-consecutive days over 7 weeks. Among these 340 speech samples, 168 were recordings of the three sustained vowels [a:], and 172 were recordings of the first two sentences of the Rainbow Passage (40 syllables). Six voice acoustic parameters were analyzed, representing a combination of traditional and contemporary metrics: alpha ratio, cepstral peak prominence (CPP), fundamental frequency (fo) mean, fo standard deviation (SD), pitch period entropy (PPE), and sound pressure levels SD. All acoustic analyses were performed using custom MATLAB scripts primarily for file handling and integrated Praat scripts (v.6.4.01, www.fon.hum.uva.nl/praat) [29]. Prior to analysis with Praat, samples were normalized but were otherwise unaltered. Within Praat are several methods of parameter extraction, along with variables that will affect the calculation. In this case, for repeatability, only default parameters and extraction variables were used. All parameters except the PPE and alpha ratio were obtained from the built-in Praat “Voice” function [30]. The alpha ratio was calculated as the energy between 1000 Hz and 5000 Hz divided by the energy between 50 Hz and 1000 Hz [31,32].

### 2.4. Statistical Analysis

First, we calculated mean values and assessed differences in voice acoustic parameters, depending on the recording instrument. Second, we assessed the agreement [33] between microphone-derived and accelerometer-derived voice acoustic parameters from the reading and vowel productions using the Bland–Altman plot [34,35]. Third, we used generalized estimating equations (GEEs) to examine the associations between the voice acoustic parameters with the moment of the recording (pre vs. post WRT) and the type of speech production (reading vs. vowel).

In addition to Bland–Altman plots, we employed generalized estimating equations (GEEs) to analyze the data. GEEs were chosen because they are particularly suitable for analyzing repeated measures or clustered data, where observations within the same subject or cluster are likely to be correlated. In our study, multiple voice recordings were taken from the same subject, leading to correlated data that GEEs can effectively manage. In addition, GEEs allow for the specification of different correlation structures, providing flexibility to model the within-subject correlation appropriately. This flexibility was crucial in accurately capturing the correlation patterns in our data. By employing GEEs, we ensured that the analysis appropriately accounted for the correlated nature of the data, providing reliable and valid estimates of the effects of interest.

We employed several methods to control potential confounding variables, ensuring the validity of our results. First, potential confounding variables (speech production and moment of the recording) were identified based on prior research and theoretical considerations. Then, we used multivariate models to statistically adjust for identified confounding variables. By including these variables as covariates in our models, we were able to isolate the effect of the recording method on the voice acoustic parameters.

## 3. Results

### 3.1. Differences in Parameters Obtained from the MIC Signal and the ACC Signal

Table 1 shows the results of the mean values and assessed differences for voice acoustic parameters depending on the recording instrument. As shown in the Table, the alpha ratio was statistically higher when recorded with the microphone in the reading sample (rainbow passage); however, when recorded from the vowel sample, it was higher when acquired with the accelerometer. Sound pressure levels standard deviations (SPL SDs) were statistically smaller for both reading and vowel production when recorded with the microphone, compared with the accelerometer. Cepstral peak prominence was statistically smaller when recorded with the microphone from the reading sample. Pitch period entropy was significantly bigger when acquired from the microphone signal, compared with the accelerometer signal, in the vowel sample.

### 3.2. Agreement Between Parameters Obtained from the MIC Signal and the ACC Signal

**Figure 2.**
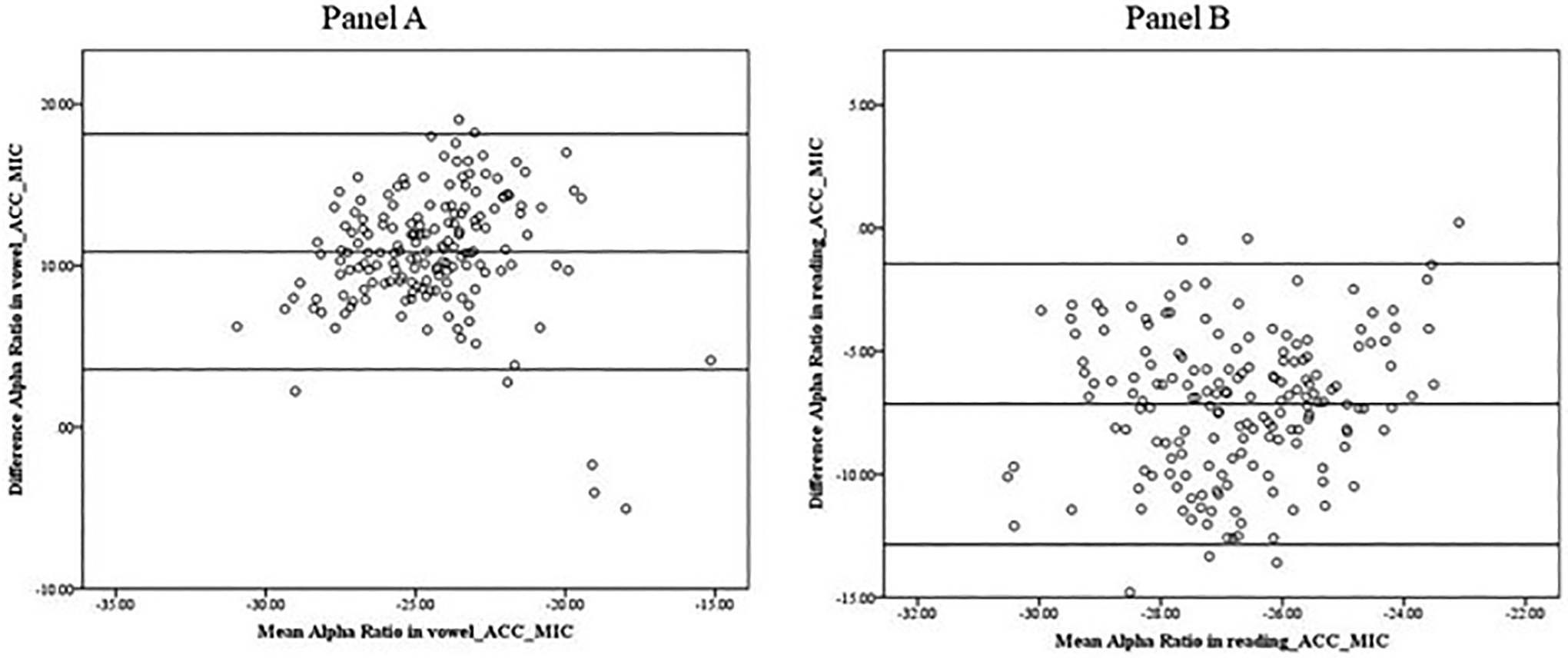
Bland–Altman plot. Data indicate that the alpha ratios obtained from the microphone signal and the accelerometer signal in the vowel production (Panel A) and the reading (Panel B) show no systematic difference in the mean scores.

Figure 2 presents Bland–Altman (B&A) plots assessing the agreement between the microphone (MIC) and accelerometer (ACC) in measuring the alpha ratio during vowel production (panel A) and reading (panel B). During reading, the average alpha ratio from the accelerometer signal was slightly lower (−30.33), compared to the microphone signal (−23.19). On the contrary, during vowel production, the average alpha ratio from the accelerometer signal was slightly higher (−19.05), compared to the microphone signal (−29.90). The B&A plots indicate mean differences of 10.86 (SD = 3.72) in vowel production and −7.15 (SD = 2.91) during reading between the microphone and accelerometer signals. These results suggest no systematic bias (ACC-MIC).

**Figure 3.**
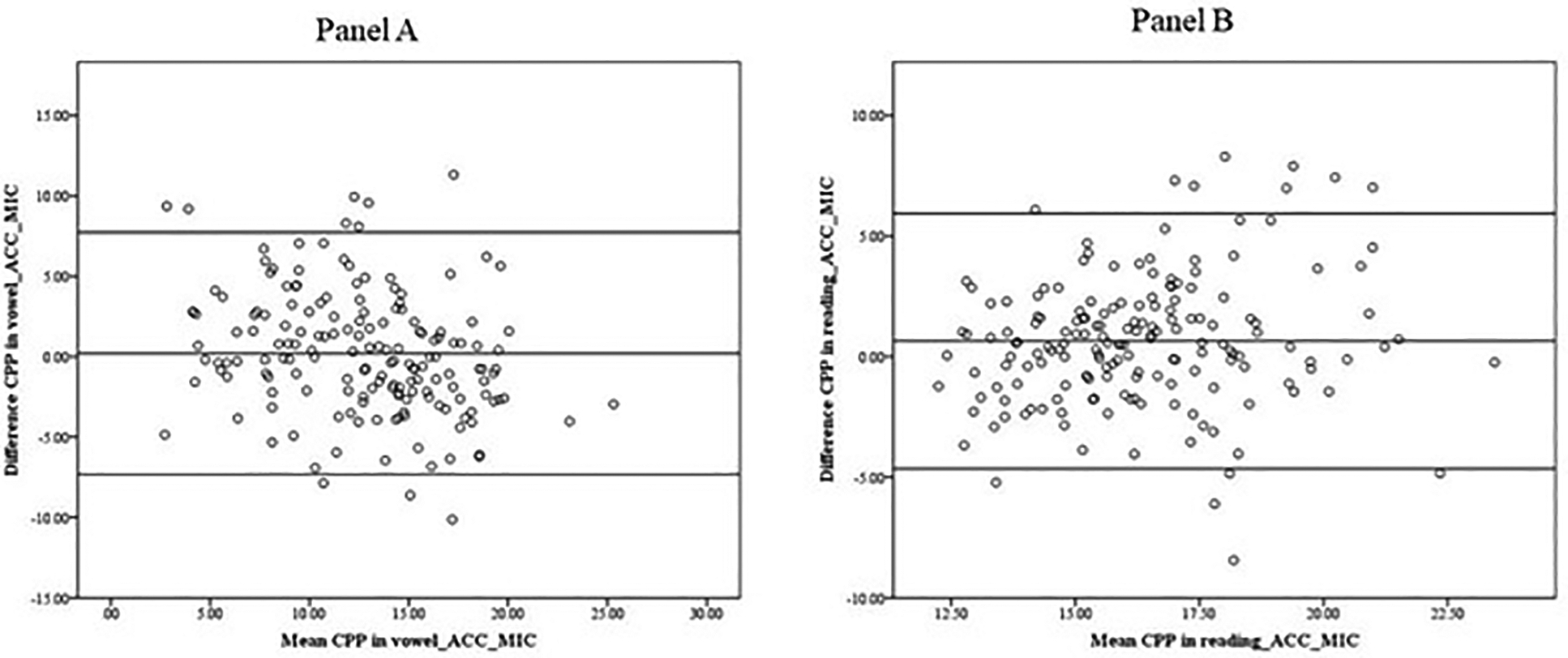
Bland–Altman plot. Data indicate that the CPPs obtained from the microphone signal and the accelerometer signal in the vowel (**Panel A**) and the reading (**Panel B**) show no systematic difference in the mean scores.

The B&A plots used to assess agreement between the CPP determined by the microphone and accelerometer in vowel production (Figure 3A) and reading (Figure 3B) indicated that although the mean CPP calculated from the accelerometer signal was slightly bigger (16.62 for reading and 12.83 in vowel) than the CPP calculated from the microphone signal (15.97 in reading and 12.63 in vowel), there was not a systematic effect of the mean values (ACC-MIC) on the difference (ACC-MIC). The mean CPP differences between the microphone and accelerometer were 0.65 (SD = 2.70) for reading and 0.20 (SD = 3.84) for vowel production.

**Figure 4.**
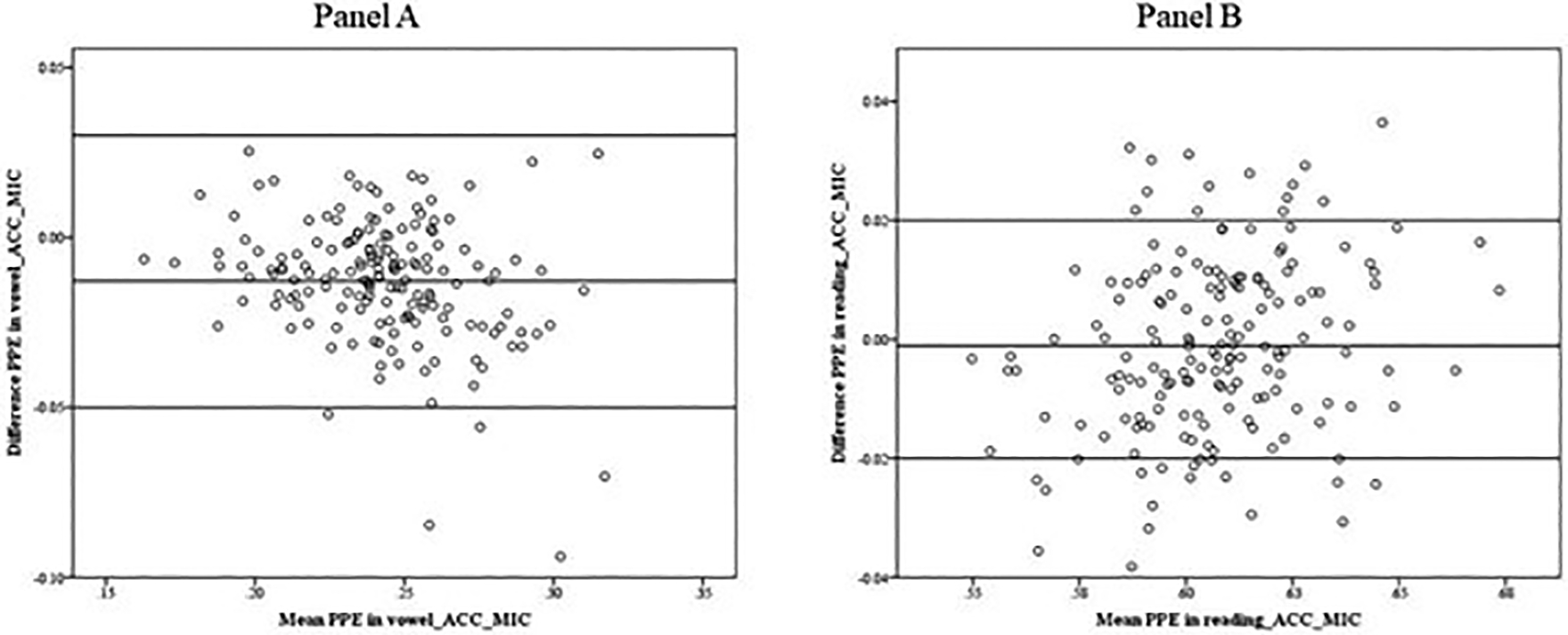
Bland–Altman plot. Data indicate that PPEs obtained from the microphone signal and the accelerometer signal in the vowel (**Panel A**) and the reading (**Panel B**) show no systematic difference in the mean scores.

Figure 4 shows the B&A plots used to assess the agreement between the pitch period entropy (PPE) determined by the microphone and the accelerometer signals in the vowel production (panel A) and the reading (panel B). In the reading, the mean PPEs calculated from the accelerometer and the microphone signal were the same (0.61), and for the vowel, the accelerometer was slightly smaller, 80.249, than the microphone (0.25). The B&A plot suggests that there was not a systematic effect of the mean values (ACC-MIC) on the difference (ACC-MIC). The averages of the differences between the microphone and accelerometer were 0.00 for the reading and −0.01 for the vowel (SD = 0.01 and 0.02, respectively).

**Figure 5.**
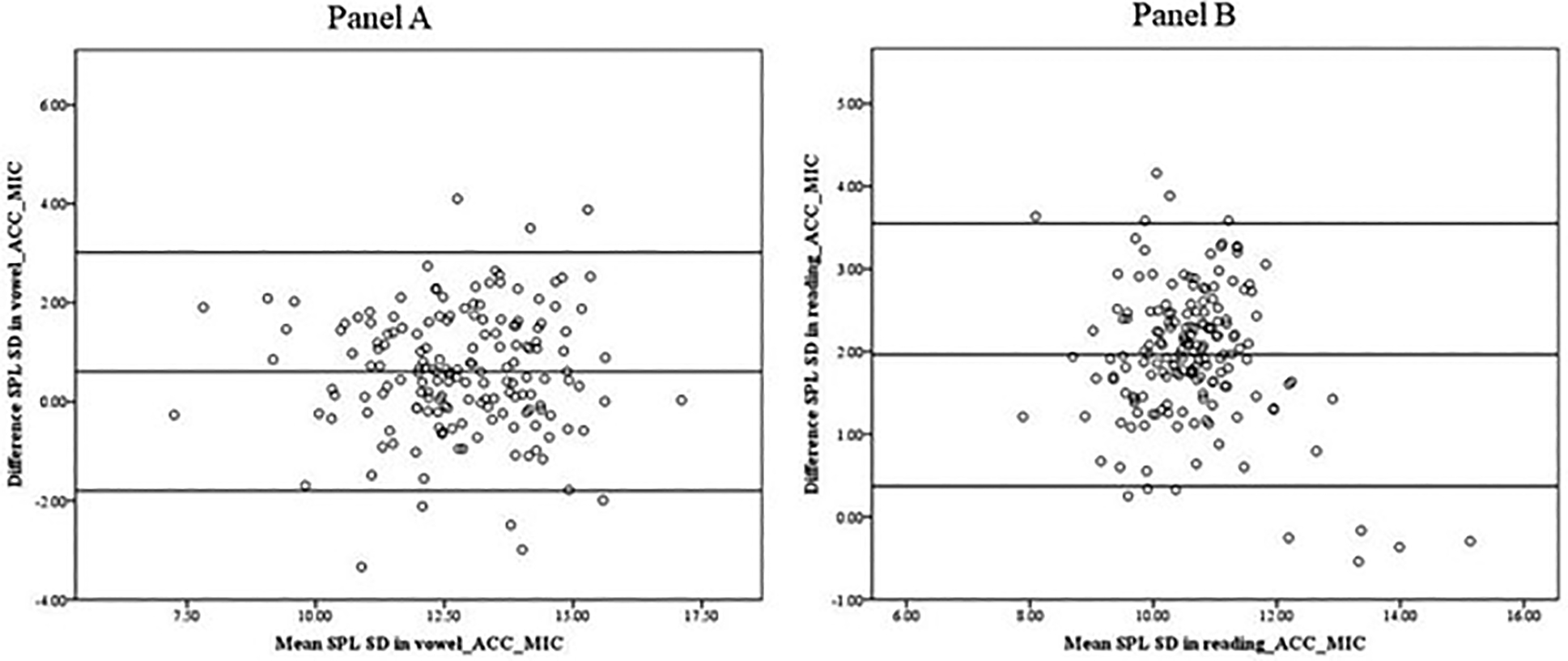
Bland–Altman plot. Data indicate that the SPL SDs obtained from the microphone signal and the accelerometer signal in the vowel (**Panel A**) and the reading (**Panel B**) show no systematic difference in the mean scores.

Figure 5 shows the B&A plot used to assess the agreement between the sound pressure levels standard deviation (SPL SD) determined by the microphone and the accelerometer signals in the vowel production (Panel A) and the reading (Panel B). While the mean SPL SD calculated from the accelerometer signal was slightly bigger (11.58 for reading, and 13.12 for vowel) than the one calculated from the microphone signal (9.62 for reading, and 12.51 for vowel), the B&A plots suggest that there was not a systematic effect of the mean values (ACC-MIC) on the difference (ACC-MIC). Therefore, any systematic bias (for instance, due to the calibration of the instruments) was constant across all measurement values and not systematic. The averages of the differences between the microphone and accelerometer were 0.61 (SD = 1.23) for vowel production and 1.96 for reading (SD = 0.81).

**Figure 6.**
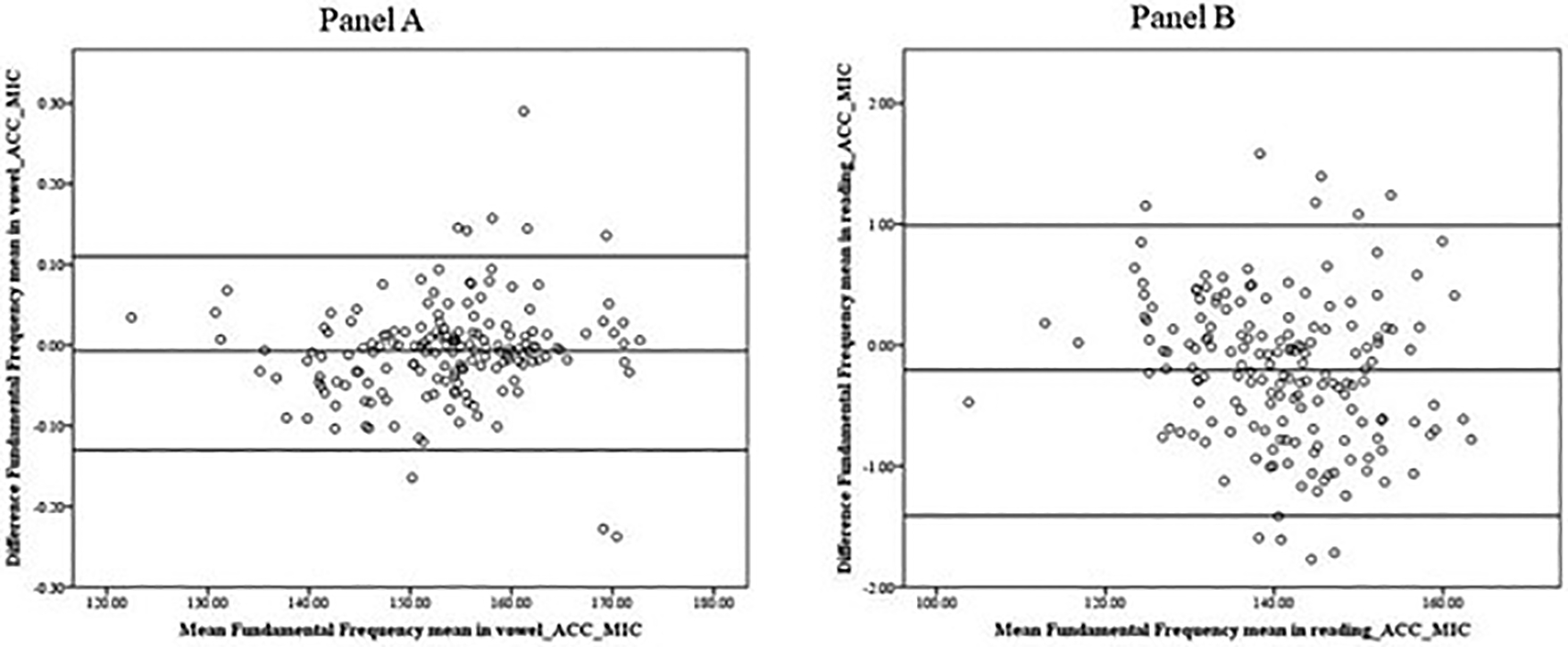
Bland–Altman plot. Data indicate that the fo means obtained from the microphone signal and the accelerometer signal in the vowel (**Panel A**) and the reading (**Panel B**) show no systematic differences in the mean scores.

For the fo mean, the averages of the differences between the microphone and accelerometer signals were −0.21 (SD = 0.61) for reading and −0.01 (SD = 0.06) for vowel production, being marginally higher in the microphone signal (140.86Hz, SD = 10Hz) than the one from the accelerometer signal (140.65, SD of 9.89). The B&A plots show a good agreement across the complete range of observed values without a systematic effect of the mean values (ACC-MIC) on the difference (ACC-MIC) in the vowel productions (Figure 6A) and reading (Figure 6B).

**Figure 7.**
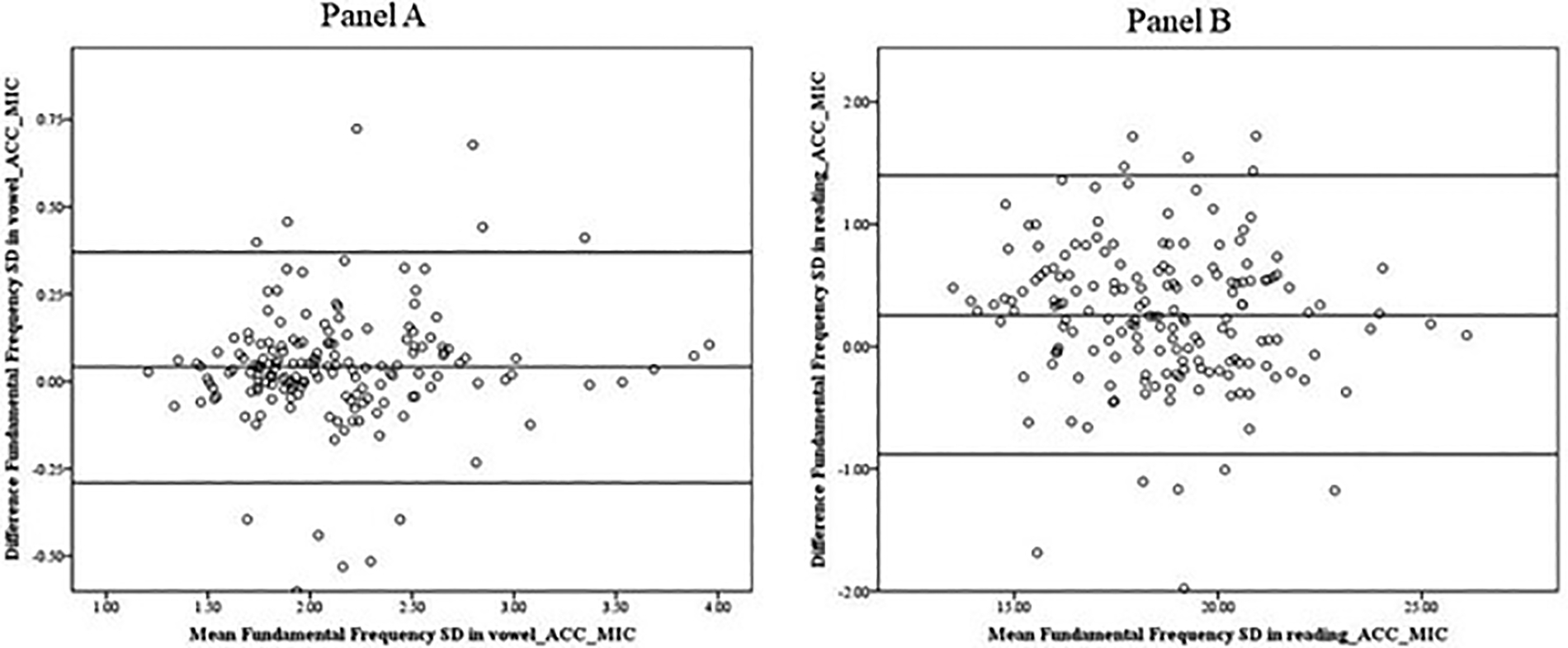
Bland–Altman plot. Data indicate that the fo SDs obtained from the microphone signal and the accelerometer signal in the vowel (**Panel A**) and the reading (**Panel B**) show a systematic difference in the mean scores.

Figure 7 shows that, for fundamental frequency standard deviation (fo SD), the decrease in the difference between the two instruments was not associated with an increase in the average mean (ACC-MIC). Therefore, any systematic bias (for instance, due to the calibration of the instruments) was constant across all measurement values. The mean fo SD calculated from the accelerometer signal was slightly higher (18.87 in reading and 2.15 in vowel), compared with the microphone signal (18.48 for reading and 2.11 for vowel production). The averages of the differences between the microphone and accelerometer signals were 0.26 (SD = 0.58) for reading and 0.04 (SD = 0.17) for vowel production.

### 3.3. Associations Between the Parameters Obtained from the MIC Signal and the ACC Signal with the Moment of the Recording and the Type of Speech Production

Table 2 shows the betas and standard errors from the GEE analysis used to examine the relationship between voice acoustic parameters obtained from the microphone signal and the accelerometer signal with those factors that were found to be associated with the voice acoustic parameters measured using the microphone and the accelerometer signals.

For the alpha ratio, multivariate analysis indicated that the type of speech (reading vs. vowel) and the moment of measurement (pre vs. post WRT) were statistically significant factors associated with this parameter. However, no effect was observed based on the instrument used (accelerometer vs. microphone). The results of the multivariate analysis suggested that the CPP was slightly smaller when calculated from the signal acquired from the microphone (B= −0.06) but larger when calculated from the reading sample (B = 0.34), compared to measurements obtained from the accelerometer and vowel production.

Fundamental frequency standard deviation was statistically smaller (B= −0.01) when calculated from the signal acquired from the microphone and larger when calculated from reading production (B = 2.08), compared to the accelerometer signal and vowel production. Three voice acoustic parameters (PPE, SPL SD, and fo mean) showed statistical associations at the univariate level with speech type, but no association was observed with the instrument (accelerometer vs. microphone).

### 3.4. Direct Spectral Comparison

**Figure 8.**
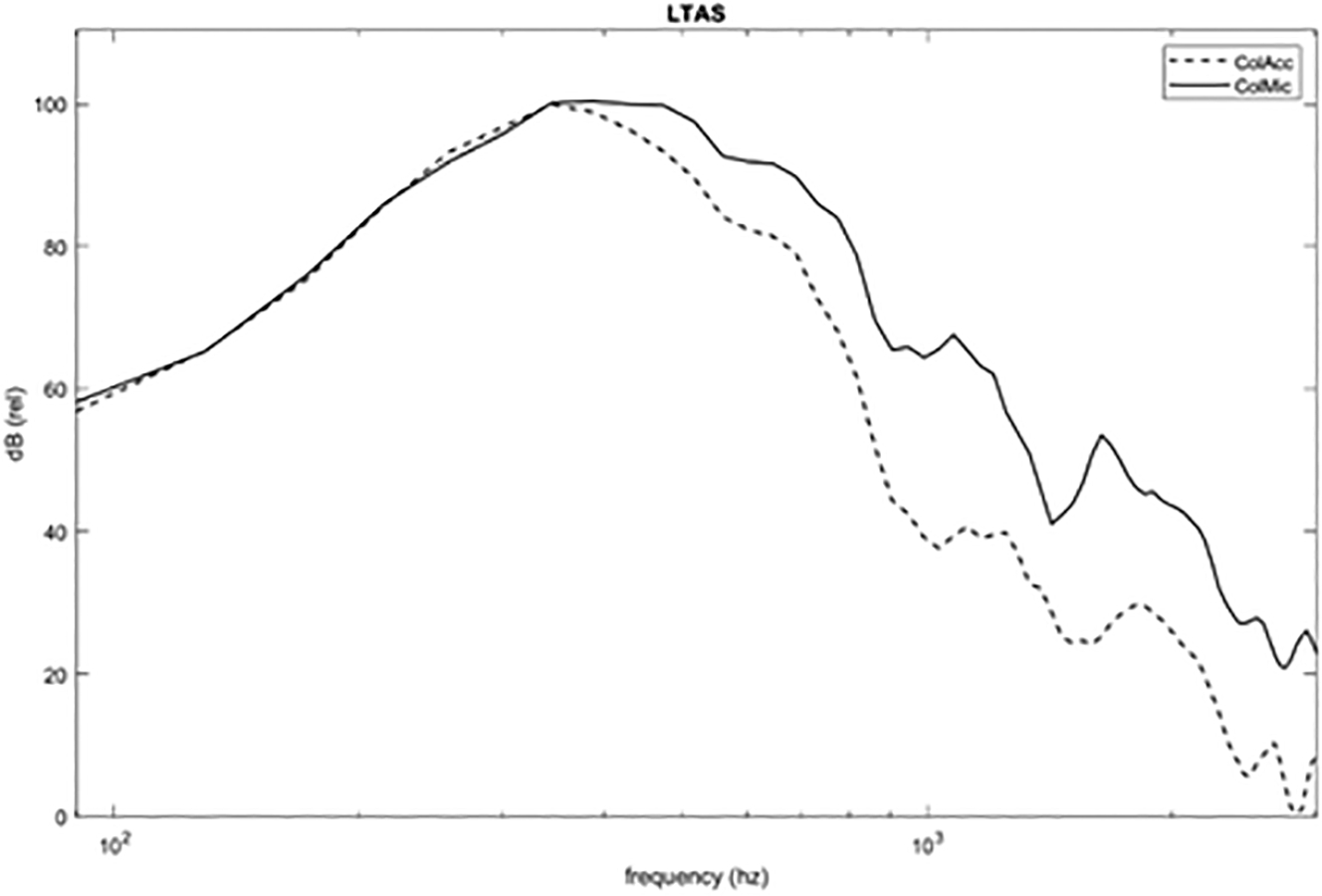
Maximum energy spectral plot of the neck-worn accelerometer (ColAcc) and microphone (ColMic).

To directly compare the spectral characteristics of the two transducers, additional recordings were collected, which included some of the same tasks as presented above, as well as some pitch glides, all produced at a range of effort levels and loudness. The recordings from each were spectrally analyzed with the maximum energy in each spectral bin calculated. Figure 8 shows the overall maximum spectral response from the voice between 90–3000Hz. Generally speaking, the two transducers matched for the range where fundamental speaking frequency was most likely to occur (<300 Hz). For higher frequencies, the accelerometer was between 10–20 dB lower than the collar microphone. It was assumed that a microphone in front of the person and not below the chin on the neck may have a slightly more subtle spectral slope, as higher frequencies will not diffract as much as lower frequencies.

## 4. Discussion

This 7-week follow-up study aimed to support our understanding of monitoring voice production in more natural settings and assessed the agreement between microphone-derived and accelerometer-derived voice acoustic parameters. In addition, we examined whether the voice acoustic parameters obtained from both signals showed similar associations with the moment of the recording (pre vs. post WRT) and type of speech production (reading vs. vowel). Five important results were found. First, Bland–Altman plots revealed no significant systematic bias (mean difference) between microphone (MIC) and accelerometer (ACC) measurements for the alpha ratio, CPP, pitch period entropy (PPE), sound pressure level standard deviation (SPL SD), or fundamental frequency (fo) mean and standard deviation (SD). Second, speech type (reading vs. vowel) and measurement timing (pre vs. post WRT) were significantly associated with the alpha ratio, while the instrument (accelerometer vs. microphone) was not. Third, microphone measurements showed a slightly lower CPP compared to the accelerometer, with reading samples producing a higher CPP compared to the steady vowels. Fourth, PPE, SPL SD, and fo mean showed significant associations with speech type (reading vs. vowel) based on univariate analysis. Fifth, microphone measurements yielded a statistically smaller fo SD compared to the accelerometer, while reading productions had a larger fo SD than vowel productions.

Voice parameters can vary significantly depending on the time of day, due to factors such as vocal fatigue, hydration levels, and circadian rhythms. To account for this variability, we compared measures recorded at similar moments of the day. This approach helped to minimize the impact of diurnal variations on our results.

Concerning the different types of speech (read speech and sustained vowel), we included both speech tasks to capture a comprehensive range of voice parameters. By analyzing both sustained vowel and read speech, we were able to account for the variability introduced by different speech contexts. Additionally, we used statistical methods to control for speech type in our analyses, ensuring that the effects of the recording method were not confounded by the type of speech produced.

The fo mean was the parameter least influenced by the type of transducer, compared to the CPP and alpha ratio. This was expected, as the fundamental frequency should be prominent in both signals (as was illustrated in Figure 8) and resilient to the tissue transmission loss of higher frequency vibration and the reduced frequency response of the accelerometer, as well as the added acoustic reflections within the airway detected by the microphone signal. Our results agreed with previous research that reported a strong correlation between the fo measured from a microphone signal and an accelerometer signal among speakers with and without dysphonia [36]. However, studies have shown that talker factors, such as sex as a biological factor, intrasubject variability, and microphone type, significantly affected fundamental frequency estimates, which could affect the agreement between instruments, depending on the fo range of the individual, e.g., very high-pitched voice [37].

Regarding our results on CPP, considering that the CPP is a measure of periodicity within a spectrum (e.g., the more harmonic a spectrum, the greater the cepstral peak is), it was expected that the microphone and accelerometer values would be similar, since performing the Fourier transform two times (once from time to frequency, then from frequency to quefrency) would mitigate some of the high-fidelity components in the microphone signal that the accelerometer signal does not have. Our results on CPP values were similar to those of Mehta et al. (2016), who reported a strong moderate correlation (r ≤ 0.90) between accelerometer and microphone signals [13]. Indirect comparable results were also reported by Bottalico et al. (2020), who found that the CPP metric was a robust parameter across six different microphones, with an average compatibility index of 0.8 [21].

Concerning the results on the alpha ratio, the spectral transfer function of the accelerometer was not sensitive to reverberant/vocal tract resonance energy, whereas the microphone had a flatter spectral transfer function [17,18]. This difference should elicit a difference in the calculation of the alpha ratio, with the accelerometer largely insensitive to strong spectral enhancement from resonant voice techniques, which would be captured by the microphone signal.

With any study, there are limitations and future opportunities. In this study, the placement of the neck collar could have been different for each recording. These differences could affect both signals, likely affecting the accelerometer signal more. At the same time, this was intentionally not controlled to allow for real-world variability to be introduced, allowing for the results to be more translatable to actual clinical situations. Further, this was a single biological male with an average fundamental frequency of about 130 Hz. With the microphone being omnidirectional (more flat transfer function) [18] and the accelerometer not having a flat transfer function, differences in average fundamental frequency could have some slight impact on the parameter values, just as using the same microphone for different people would. Nevertheless, the high quantity of repeated measures over time showed that the results were highly robust, even with these limitations.

Additionally, while this study demonstrated the robustness of neck-worn accelerometers, it only evaluated one type of accelerometer and microphone. Future research should explore whether other contact microphones, including throat microphones, could yield similar or more accessible results for researchers and clinicians. Testing lower-cost options could help broaden the utility of such devices, even in other contexts, such as clinical environments with high background noise (e.g., critical care units, outpatient clinics, or bedside assessments).

The authors would like to mention the potential benefits of incorporating machine learning methods into the agreement assessment. While this approach is beyond the current scope of our manuscript, we recognize its value and have identified it as a promising direction for future research. This consideration underscores the evolving nature of our work and the potential for advanced methodologies to enhance our evaluation processes in subsequent studies.

It is also important to acknowledge that the results may not be fully generalizable to all settings, including occupational environments with distinct noise profiles, such as classrooms or call centers. The influence of various noise types (e.g., low- vs. high-frequency noise) on voice assessment was not explored in this study [38,39]. Future research should address these gaps by testing devices across diverse occupational and clinical settings and including individuals with varying vocal profiles, such as female participants, to enhance the applicability of these findings.

## 5. Conclusions

There is a need to better understand ecological voice assessment, which may lead to more practical and/or efficient clinical assessment protocols. The findings of this study demonstrated that the fundamental frequency, alpha ratio, PPE, and SPL SD values are robust, regardless of the instrument used, whether it be a microphone or a neck-worn accelerometer. This robustness suggests that accelerometers can be effectively utilized in less-controlled environments, providing a reliable alternative to traditional microphones for voice assessments. The practical implications of these results are significant for clinical settings, as they offer a more flexible and accessible means of monitoring vocal health.

By validating the use of accelerometers, clinicians can confidently employ these devices in various real-world scenarios, such as remote therapy and ecological voice assessments. This approach not only enhances the accuracy of vocal health evaluations but also allows for continuous monitoring of patients in their natural environments. Consequently, the integration of accelerometers into clinical practice can lead to more personalized and effective intervention strategies, ultimately improving patient outcomes.

In summary, the study supports the adoption of neck-worn accelerometers, and possibly throat or contact microphones, as a viable tool for voice assessment, paving the way for more comprehensive and ecologically valid evaluations of vocal function.

## Figures and Tables

**Table 1.** Mean values of voice acoustic parameters per speech sample and recording transducer.

Parameter	Reading Sample		Vowel Sample	
	ACC	MIC	ACC	MIC
Alpha Ratio *	−30.33	−23.19	−19.05	−29.90
Cepstral Peak Prominence ¥	16.62	15.97	12.83	12.63
Pitch Period Entropy ±	0.61	0.61	0.24	0.25
Sound Pressure Level SDs *	11.58	9.62	13.12	12.51
Fundamental Frequency mean	140.65	140.86	153.52	153.52
Fundamental Frequency SD	18.74	18.48	2.15	2.11

SD = Standard deviation;

* statistically significant differences in both reading and vowel samples;

¥ statistically significant differences in reading samples;

± statistically significant differences in vowel samples.

**Table 2.** Generalized estimating equations (GEEs) to investigate associations between the voice acoustic parameters with the type of instrument (microphone vs. accelerometer), type of speech (vowel vs. reading), and moment of the measurement (pre vs. post).

	Univariate Analysis			Multivariate Analysis		
CPP	Beta	SE	*p*-value	Beta	SE	*p*-value
Accelerometer	Reference Category			Reference Category		
Microphone	−0.11	0.06	0.05	−0.06	0.03	0.05
Sustained vowel	Reference Category			Reference Category		
Rainbow passage	0.32	0.06	0.00	0.34	0.07	0.00
Measure before the WRT	Reference Category			Reference Category		
Measure after the WRT	0.19	0.12	0.10	0.05	0.04	0.29
Alpha Ratio	Beta	SE	*p*-value	Beta	SE	*p*-value
Accelerometer	Reference Category			Reference Category		
Microphone	2.99	1.50	0.05	1.45	0.79	0.07
Sustained vowel	Reference Category			Reference Category		
Rainbow passage	−3.90	0.79	0.00	−3.42	0.54	0.00
Measure before the WRT	Reference Category			Reference Category		
Measure after the WRT	1.30	0.30	0.00	1.09	0.15	0.00
fo SD	Beta	SE	P-value	Beta	SE	P-value
Accelerometer	Reference Category			Reference Category		
Microphone	−0.01	0.00	0.00	−0.01	0.00	0.00
Sustained vowel	Reference Category			Reference Category		
Rainbow passage	2.08	0.06	0.00	2.08	0.06	0.00
Measure before the WRT	Reference Category					
Measure after the WRT	−0.02	0.02	0.32			
SPL SD	Beta	SE	P-value	Beta	SE	P-value
Accelerometer	Reference Category					
Microphone	−0.03	0.07	0.65			
Sustained vowel	Reference Category					
Rainbow passage	1.10	0.02	0.00			
Measure before the WRT	Reference Category					
Measure after the WRT	0.01	0.07	0.85			
Fo	Beta	SE	P-value	Beta	SE	P-value
Accelerometer	Reference Category					
Microphone	0.04	0.04	0.41			
Sustained vowel	Reference Category					
Rainbow passage	−0.23	0.06	0.00			
Measure before the WRT	Reference Category					
Measure after the WRT	0.00	0.03	0.93			
PPE	Beta	SE	P-value	Beta	SE	P-value
Accelerometer	Reference Category					
Microphone	0.03	0.03	0.33			
Sustained vowel	Reference Category					
Rainbow passage	0.84	0.03	0.00			
Measure before the WRT	Reference Category					
Measure after the WRT	0.00	0.04	0.98			

## Data Availability

We encourage all authors of articles published in MDPI journals to share their research data. In this section, please provide details regarding where data supporting reported results can be found, including links to publicly archived datasets analyzed or generated during the study. Where no new data were created, or where data are unavailable due to privacy or ethical restrictions, a statement is still required. Suggested Data Availability Statements are available in section “MDPI Research Data Policies” at https://www.mdpi.com/ethics.

## References

[1] Patel RR; Awan SN; Barkmeier-Kraemer J; Courey M; Deliyski D; Eadie T; Paul D; Švec JG; Hillman R Recommended Protocols for Instrumental Assessment of Voice: American Speech-Language-Hearing Association Expert Panel to Develop a Protocol for Instrumental Assessment of Vocal Function. Am. J. Speech Lang. Pathol 2018, 27, 887–905. 10.1044/2018_AJSLP-17-0009. 29955816

[2] Dejonckere PH; Bradley P; Clemente P; Cornut G; Crevier-Buchman L; Friedrich G; Van De Heyning P; Remacle M; Woisard V A Basic Protocol for Functional Assessment of Voice Pathology, Especially for Investigating the Efficacy of (Phonosurgical) Treatments and Evaluating New Assessment Techniques. Eur. Arch. Oto-Rhino-Laryngol 2001, 258, 77–82. 10.1007/s004050000299. 11307610

[3] Maryn Y; Corthals P; Van Cauwenberge P; Roy N; De Bodt M Toward Improved Ecological Validity in the Acoustic Measurement of Overall Voice Quality: Combining Continuous Speech and Sustained Vowels. J. Voice 2010, 24, 540–555. 10.1016/j.jvoice.2008.12.014. 19883993

[4] Maryn Y; De Bodt M; Roy N The Acoustic Voice Quality Index: Toward Improved Treatment Outcomes Assessment in Voice Disorders. J. Commun. Disord 2010, 43, 161–174. 10.1016/j.jcomdis.2009.12.004. 20080243

[5] Castillo-Allendes A; Cantor-Cutiva LC; Hunter EJ Acoustic Effects of Vocal Warm-Up: A 7-Week Longitudinal Case Study. J. Voice 2024, 38, 458–465. 10.1016/j.jvoice.2021.09.030. 34844825 PMC9133272

[6] Ferguson SH; Morgan SD; Hunter EJ Within-Talker and within-Session Stability of Acoustic Characteristics of Conversational and Clear Speaking Styles. J. Acoust. Soc. Am 2024, 155, 44–55. 10.1121/10.0024241. 38174965 PMC10990565

[7] Lowell SY The Acoustic Assessment of Voice in Continuous Speech. Perspect. Voice Voice Disord 2012, 22, 57–63. 10.1044/vvd22.2.57.

[8] da Silva W; Constantini AC Speech Task Affects the Objective Evaluation of Dysphonic Voices. J. Speech Sci 2018, 7, 1–15. 10.20396/joss.v7i1.14988.

[9] Vogel AP; Fletcher J; Snyder PJ; Fredrickson A; Maruff P Reliability, Stability, and Sensitivity to Change and Impairment in Acoustic Measures of Timing and Frequency. J. Voice 2011, 25, 137–149. 10.1016/j.jvoice.2009.09.003. 20171828

[10] Castellana A; Carullo A; Astolfi A; Puglisi GE; Fugiglando U Intra-Speaker and Inter-Speaker Variability in Speech Sound Pressure Level across Repeated Readings. J. Acoust. Soc. Am 2017, 141, 2353–2363. 10.1121/1.4979115. 28464626

[11] Švec JG; Titze IR; Popolo PS Estimation of Sound Pressure Levels of Voiced Speech from Skin Vibration of the Neck. J. Acoust. Soc. Am 2005, 117, 1386–1394. 10.1121/1.1850074. 15807026

[12] Mehta DD; Zañartu M; Feng SW; Cheyne HA II; Hillman RE Mobile Voice Health Monitoring Using a Wearable Accelerometer Sensor and a Smartphone Platform. IEEE Trans. Biomed. Eng 2012, 59, 3090–3096. 10.1109/TBME.2012.2207896. 22875236 PMC3539821

[13] Mehta DD; Van Stan JH; Hillman RE Relationships between Vocal Function Measures Derived from an Acoustic Microphone and a Subglottal Neck-Surface Accelerometer. IEEE/ACM Trans. Audio Speech Lang. Process 2016, 24, 659–668. 10.1109/TASLP.2016.2516647. 27066520 PMC4826073

[14] Bottalico P; Ipsaro Passione I; Astolfi A; Carullo A; Hunter EJ Accuracy of the Quantities Measured by Four Vocal Dosimeters and Its Uncertainty. J. Acoust. Soc. Am 2018, 143, 1591–1602. 10.1121/1.5027816. 29604673 PMC5864503

[15] Coleman RF Comparison of Microphone and Neck-Mounted Accelerometer Monitoring of the Performing Voice. J. Voice 1988, 2, 200–205. 10.1016/S0892-1997(88)80077-8.

[16] Zanartu M; Ho JC; Kraman SS; Pasterkamp H; Huber JE; Wodicka GR Air-Borne and Tissue-Borne Sensitivities of Bioacoustic Sensors Used on the Skin Surface. IEEE Trans. Biomed. Eng 2009, 56, 443–451. 10.1109/TBME.2008.2008165. 19272887

[17] Popolo PS; Švec JG; Titze IR Adaptation of a Pocket PC for Use as a Wearable Voice Dosimeter. J. Speech Lang. Hear. Res 2005, 48, 780–791. 10.1044/1092-4388(2005/054). 16378473

[18] Hunter EJ; Spielman J; Starr A; Popolo P Acoustic Voice Recording, “I Am Seeking Recommendations for Voice Recording Hardware…”. Perspect. Voice Voice Disord 2007, 17, 7–14. 10.1044/vvd17.3.7.

[19] Castellana A; Carullo A; Casassa F; Astolfi A; Pavese L; Puglisi G Performance comparison of different contact microphones used for voice monitoring. In Proceedings of the International Congress on Sound and Vibration, Florence, Italy, 12–16 July 2015.

[20] van der Woerd B; Wu M; Parsa V; Doyle PC; Fung K Evaluation of Acoustic Analyses of Voice in Nonoptimized Conditions. J. Speech Lang. Hear. Res 2020, 63, 3991–3999. 10.1044/2020_JSLHR-20-00212. 33186510

[21] Bottalico P; Codino J; Cantor-Cutiva LC; Marks K; Nudelman CJ; Skeffington J; Shrivastav R; Jackson-Menaldi MC; Hunter EJ; Rubin AD Reproducibility of Voice Parameters: The Effect of Room Acoustics and Microphones. J. Voice 2020, 34, 320–334. 10.1016/j.jvoice.2018.10.016. 30471944 PMC6529301

[22] Hillman RE; Heaton JT; Masaki A; Zeitels SM; Cheyne HA Ambulatory Monitoring of Disordered Voices. Ann. Otol. Rhinol. Laryngol 2006, 115, 795–801. 10.1177/000348940611501101. 17165660

[23] Buekers R; Bierens E; Kingma H; Marres EHMA Vocal Load as Measured by the Voice Accumulator. Folia Phoniatr. Logop 2009, 47, 252–261. 10.1159/000266359. 8563777

[24] Ghassemi M; Van Stan JH; Mehta DD; Zañartu M; Cheyne HA; Hillman RE; Guttag JV Learning to Detect Vocal Hyperfunction from Ambulatory Neck-Surface Acceleration Features: Initial Results for Vocal Fold Nodules. IEEE Trans. Biomed. Eng 2014, 61, 1668–1675. 10.1109/TBME.2013.2297372. 24845276 PMC4077201

[25] Deliyski DD; Shaw HS; Evans MK Adverse Effects of Environmental Noise on Acoustic Voice Quality Measurements. J. Voice 2005, 19, 15–28. 10.1016/j.jvoice.2004.07.003. 15766847

[26] Bottalico P Speech Adjustments for Room Acoustics and Their Effects on Vocal Effort. J. Voice 2017, 31, 392.e1–392.e12. 10.1016/j.jvoice.2016.10.001. PMC5409880 28029555

[27] Whitling S; Rydell R; Lyberg Åhlander V Design of a Clinical Vocal Loading Test With Long-Time Measurement of Voice. J. Voice 2015, 29, 261.e13–261.e27. 10.1016/j.jvoice.2014.07.012. 25499518

[28] Fairbanks G The Rainbow Passage. In Voice and Articulation Drillbook; Harper & Row: New York, NY, USA, 1960; p. 127.

[29] Boersma P; Weenink D PRAAT, a System for Doing Phonetics by Computer. Glot Int 2001, 5, 341–345.

[30] Boersma P; Weenink D Voice Function Available online: https://www.fon.hum.uva.nl/praat/manual/Voice.html (accessed on 7 January 2025).

[31] Graetzer S; Bottalico P; Hunter EJ Speech Produced in Noise: Relationship between Listening Difficulty and Acoustic and Durational Parametersa). J. Acoust. Soc. Am 2017, 142, 974–983. 10.1121/1.4997906. 28863615 PMC5648561

[32] Monson BB; Hunter EJ; Story BH Horizontal Directivity of Low- and High-Frequency Energy in Speech and Singing. J. Acoust. Soc. Am 2012, 132, 433–441. 10.1121/1.4725963. 22779490 PMC3407162

[33] Bland JM; Altman DG Statistical methods for assessing agreement between two methods of clinical measurement. Lancet 1986, 327, 307–310. 10.1016/S0140-6736(86)90837-8. 2868172

[34] Myles PS; Cui JI Using the Bland–Altman Method to Measure Agreement with Repeated Measures. Br. J. Anaesth 2007, 99, 309–311. 10.1093/bja/aem214. 17702826

[35] Bland JM; Altman DG Agreement Between Methods of Measurement with Multiple Observations Per Individual. J. Biopharm. Stat 2007, 17, 571–582. 10.1080/10543400701329422. 17613642

[36] Cristina Oliveira R; Gama ACC; Magalhães MDC Fundamental Voice Frequency: Acoustic, Electroglottographic, and Accelerometer Measurement in Individuals With and Without Vocal Alteration. J. Voice 2021, 35, 174–180. 10.1016/j.jvoice.2019.08.004. 31575435

[37] Deliyski DD; Shaw HS; Evans MK; Vesselinov R Regression Tree Approach to Studying Factors Influencing Acoustic Voice Analysis. Folia Phoniatr. Logop 2006, 58, 274–288. 10.1159/000093184. 16825780

[38] Bottalico P; Astolfi A Investigations into Vocal Doses and Parameters Pertaining to Primary School Teachers in Classrooms. J. Acoust. Soc. Am 2012, 131, 2817–2827. 10.1121/1.3689549. 22501060

[39] Astolfi A; Castellana A; Puglisi GE; Fugiglando U; Carullo A Speech Level Parameters in Very Low and Excessive Reverberation Measured with a Contact-Sensor-Based Device and a Headworn Microphone. J. Acoust. Soc. Am 2019, 145, 2540–2551. 10.1121/1.5098942. 31046351

